# Implementation of a Service-Learning Project Focused on Handwashing and Vaccinations Within an Undergraduate Microbiology Laboratory Course

**DOI:** 10.3389/fmicb.2021.587094

**Published:** 2021-05-19

**Authors:** Beth A. Potter

**Affiliations:** Department of Biology, School of Science, Penn State Behrend, Erie, PA, United States

**Keywords:** service-learning, handwashing, vaccinations, microbiology education, undergraduate

## Abstract

Two relevant topics in keeping populations healthy are handwashing and vaccinations. Thus, the service-learning project titled “We Are Healthy” campaign was introduced within a microbiology laboratory course with two objectives; our biologists would better understand the importance of these actions by designing activities that engage the student community and to obtain an understanding of the campus community’s behaviors and beliefs concerning these topics. Students designed the campaign to include handwashing stations, pictures of bacterial cultures from swabbing common surfaces, and trivia questions testing their peers’ knowledge of various vaccines, as well as handwashing and vaccination surveys. To assess the impact of the campaign on microbiology students (*n* = 34), they were provided 10 questions that were scored on a scale from 1 to 5 (1 = strongly disagree; 5 = strongly agree). Student gains (score > 3) were reported for depth in knowledge, development of better public speaking skills, and greater respect for volunteers suggesting that the campaign was beneficial. This study subsequently led to the receiving of grants that allowed the continuation of the campaign within the course, the securing of funding for handwashing and hand sanitizing stations and the initiation of new undergraduate research projects.

## Introduction

It is likely that the words “experience” and “leadership” are mentioned multiple times in a typical advising appointment that is focused on career goals. Twenty-five years ago, most lectures consisted of a one-sided, continuous dialog by the instructor and laboratories were designed to provide specific results through a cookie-cutter-type design; thus, students were most likely instructed to gain experience and leadership through extracurricular formats *via* undergraduate research opportunities, jobs, and organizations/clubs. The idea of including these opportunities within the classroom was at the heart of the American Association for the Advancement of Science’s (2011) report. By embedding real research projects within classroom laboratories, all students were inevitably pushed into the driver seat of their education through the exploration of novel research questions. Active engagement within these CUREs works to build strong science foundations by focusing on students gaining a deeper understanding of the scientific process as well as analytical/technical skills (Thiry et al., 2011; Auchincloss et al., 2014). However, to provide students with the best resume for success, undergraduate experiences should not be confined by classroom/laboratory walls and should inspire biologists to become leaders not only within their field but also within their community (Hart, 2015; Webb, 2016; McGowin and Teed, 2019). This is especially important in current times where most would not disagree that there is a disconnection between scientific research and the media. Explanations for the disconnection involve a general underestimation of how difficult it is to connect with diverse audiences, perceptions that the public does not want to understand science, a lack of recognition for outreach/community engagement, and the lack of formal training in undergraduate and graduate curricula (Brownell et al., 2013; Devonshire and Hathway, 2014; Varner, 2014). Following the success of CUREs, it seems that including community engagement within the curricula could provide a new dignity within the field of biology. Service-learning projects aim to fill a need on both sides; a community need is satisfied, and the students gain a wider perspective of their course content and build a connection with their community (Bringle and Hatcher, 1996). This paper briefly describes the incorporation of a service-learning project into a microbiology laboratory course in which students developed a campaign to increase awareness toward two important factors regarding human health, handwashing, and vaccinations.

The idea for a campaign that focused on handwashing was born from many students saying they wash their hands more often after an early laboratory introduced them to the frequency of bacteria around them, the chemistry of handwashing, and a review of proper handwashing techniques. The recent coronavirus pandemic hopefully reminded us all that our hands are important transmission factors in the spread of both respiratory and gastrointestinal infections; thus, the best way to limit pathogen access to our bodies is through good hand hygiene (Drankiewicz and Dundes, 2003; Allegranzi and Pittet, 2009; Mackert et al., 2013). While handwashing is considered as a social norm in developed countries, studies suggest that proper hand hygiene is not occurring (Anderson et al., 2008). In a direct observational study of several public restrooms, only 66.9% washed their hands with soap and only 5% washed for more than 15 s as designated by the CDC (Borchgrevink et al., 2013). Vaccinations were included within this project primarily because the university was changing their policy regarding vaccinations, requiring the measles, mumps, and rubella vaccine for all degree-seeking students and the meningococcal vaccine for all students living on campus; however, this is a major medical issue as anti-vaccine campaigns continue to increase. Vaccines were once proclaimed lifesavers by parents, but the number of vaccine-hesitant parents is growing at an alarming rate. It is estimated that 15% of children are under-immunized with parents doubting the effectiveness of vaccines and having concerns regarding the amount of vaccines children are given before the age of two (Rabinowitz et al., 2016). Additionally, the decrease in immunization rates is a serious concern because it threatens the herd immunity model (Berezin and Eads, 2016; Sobo, 2016). Vaccines can only be effective if a threshold of the population is vaccinated to reduce the transmission rates of the disease lowering the incidence of the disease (Orenstein and Ahmed, 2017). As more parents are choosing to reduce immunization rates, thresholds needed within the population to maintain safety are in jeopardy.

The service-learning event was designated the “We Are Healthy” campaign playing off the popular “We Are…Penn State” phrase that is known by all current and former Penn State students. The campaign was introduced within a microbiology laboratory course with two objectives; our biologists would better understand the importance of these actions by designing activities that engage the student community and obtain an understanding of the campus community’s behaviors and beliefs concerning these topics.

## Materials and Methods

The campaign was incorporated into three sections of the MICRB202 Introduction to Microbiology laboratory course at Penn State Erie in the fall 2017 semester. In the weeks preceding the event, students were asked to do several assignments and discuss the campaign during a portion of the laboratory session. Students were asked to design handwashing advertisements stressing that the good handwashing requires 20 s of scrubbing with soap. The advertisements were then placed throughout the campus (student union eatery and bathrooms). Central to the campaign were handwashing demonstrations. For this, several portable handwashing stations were rented so proper handwashing techniques could be demonstrated using Glitterbug potion and viewing stations (Brevis Corporation). For the vaccine portion of the campaign, groups of 2–4 students were assigned one of four vaccines: MMR, meningitis, human papilloma virus, or influenza, to research and present to the class. From their research, students were asked to develop multiple-choice or true/false questions that could be used as trivia questions during the campaign. Survey questions were developed to understand participants’ views regarding handwashing and vaccinations. Handwashing surveys asked when students washed their hands after five daily activities, how long they wash their hands, four agree/disagree statements, and two questions regarding hand sanitizers for a total of 13 questions. Vaccine surveys contained five agree/disagree statements and 11 yes/no questions that focused on some general thoughts toward vaccination programs, an understanding of the term herd immunity, and some specific questions regarding the flu, measles, meningitis, and HPV vaccines (these were chosen because they are either required or highly recommended before attending college). Surveys were done on paper, and participants needed to circle their answers.

Once the main activities were determined, a strategy was needed to get passing-by students to participate in the campaign. Students wanted to use a food that required participants to use their hands rather than a utensil and decided on popcorn. It was also decided that a freebie item, a syringe pen, would be awarded to those who successfully answered a trivia question. In this strategy, a student assigned as the Go-Getter, would boldly walk up to students, ask them to fill out the surveys, and upon completion, receive a bag of popcorn. Popcorn machines were borrowed from the athletics department and student government association. Another student assigned as the survey collector would instruct students to drop their surveys into a covered box and instruct the participant that they could get their popcorn after they washed their hands. Students assigned as the handwashing police would supply Glitterbug potion and keep the time of handwashing. After properly washing their hands, students were given their popcorn from the student assigned as the popcorn distributor. The student assigned as the trivia host would then ask the student a question for their chance to win a syringe pen. Questions were presented to students in large bold print on a standard piece of paper within a page protector in which the host could read the question and the answer choices were displayed to the participant. For the campaign, microbiology students (6–10) were stationed at two high-traffic areas within our campus and were wearing T-shirts with the campaign logo. Each student had a designated role and participated in the campaign for 2 h, while the event occurred at both sites simultaneously for 4 h.

### Results and Discussion

Overall, this event was easily incorporated into an introductory laboratory and additional costs were minimal. The largest costs were purchasing coordinating T-shirts and rental of handwashing stations. To reduce these costs, the campus bathrooms could be used though the timing of 20 s could be harder to maintain. Coordinating shirts are not necessary for such a campaign, but it is helpful and could be arranged by wearing school colors or spirit shirts the students already own. The most common comment from students was that it was harder than they thought to engage with their fellow students. Popcorn and syringe pens were great incentives for engagement, and based on the amount of surveys collected, we interacted with over 400 students. The most common comment from participants was their surprise at how long 20 s is and their admission that they do not typically wash their hands for that long. Participants were also asked to respond to two separate surveys each containing more than ten questions. Due to the number of questions, the results were not completed by the end of the class so shorter surveys would be suggested. It was revealed that less than 50% of the students reported that they washed their hands for the recommended 15–20 s. Students were not surprised by this but seeing the statistics from a group familiar to them made the statistic more “real,” and we discussed how they might approach the issue in their future careers. In regard to the vaccination survey, it was shown that most of the participants believe that immunizations are an important part of a healthy lifestyle, but students were quick to point out that the size of the minority that was either neutral or disagreed (11%) with the statement was not ideal. Since participation time is limited in the described campaign, a deeper understanding of vaccination programs by participants would be more likely if the campaign was coordinated with a keynote speaker from the state or county health department.

**Figure 1:**
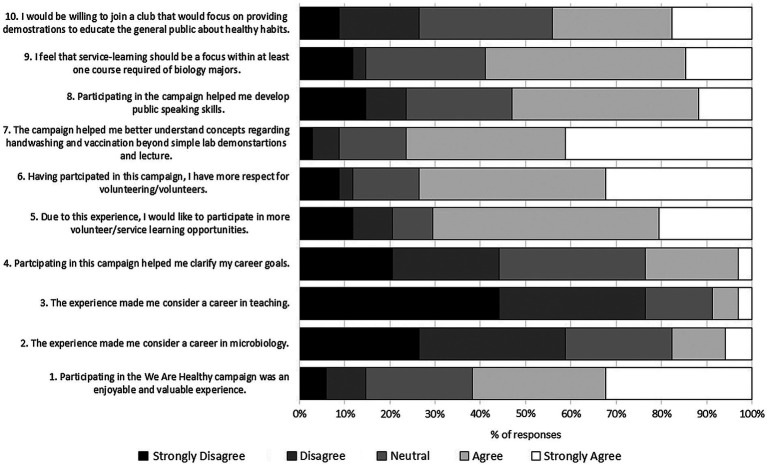
Assessment questions and responses from microbiology students (*n* = 34) after participation in the “We Are Healthy” campaign.

To assess the impact of the campaign on microbiology students (*n* = 34), they were provided ten questions which were adapted from questions asked in a published study assessing the inclusion of a service-learning project within a microbiology course (Webb, 2016). Questions were scored on a scale from 1 to 5 (1 = strongly disagree; 5 = strongly agree). Figure 1 shows student gains (score > 3) were reported for depth in knowledge, development of better public speaking skills, and greater respect for volunteers suggesting that the campaign was beneficial. Little effect was shown on student career choices which may not be surprising as this course is typically taken in their third or fourth year when they have started focusing on specific career goals. Thus, it might be helpful to include service-learning projects earlier within the curriculum, perhaps even during a program-specific first-year seminar experience.

Given the importance of handwashing and vaccinations, this service-learning project could be repeated each year. To add flexibility and allow students more control in the planning, venues could be rotated to include elementary classrooms, after-school programs, and senior care facilities. Since a significant amount of data was collected in the campaign, a compilation of the data was undertaken by several students as part of an undergraduate research experience and presented at a local Sigma Xi conference. The results of this campaign have been used to apply for a few internal grants that have allowed for the continuation of the campaign, purchase of our handwashing station to use in outreach events, an increase in the number of public hand sanitizer stations, and inspired a few students to further understand hygiene behaviors specifically within the campus workout facility.

In conclusion, embedding this service-learning project got students involved in sharing course knowledge with their immediate community in a way that was active and fun for the community *via* engaging demonstrations, trivia questions, and small incentives. The inclusion of course-based service-learning projects as well as research experiences would place experience and leadership opportunities at the core of our curricula and would provide a solid foundation for students to advance the field of biology.

## Data Availability Statement

The original contributions presented in the study are included in the article/supplementary materials, and further inquiries can be directed to the corresponding author.

## Ethics Statement

The studies involving human participants were reviewed and approved by Penn State Office for Research Protections. The patients/participants provided their written informed consent to participate in this study.

## Author Contributions

BP conceived and designed the service-learning project and wrote the manuscript.

### Conflict of Interest

The author declares that the research was conducted in the absence of any commercial or financial relationships that could be construed as a potential conflict of interest.
